# Epidemiology of Constipation in Elderly People in Parts of China: A Multicenter Study

**DOI:** 10.3389/fpubh.2022.823987

**Published:** 2022-06-15

**Authors:** Xiaoshan Du, Shuai Liu, Peifei Jia, Xiaodan Wang, Jinghuan Gan, Wenzheng Hu, Han Zhu, Yehua Song, Jianping Niu, Yong Ji

**Affiliations:** ^1^Clinical College of Neurology, Neurosurgery and Neurorehabilitation, Tianjin Medical University, Tianjin, China; ^2^Tianjin Key Laboratory of Cerebrovascular and of Neurodegenerative Diseases, Department of Neurology, Tianjin Dementia Institute, Tianjin Huanhu Hospital, Tianjin, China; ^3^Department of Neurology, The Second Affiliated Hospital of Baotou Medical College, Baotou, China; ^4^Department of Neurology, China National Clinical Research Center for Neurological Diseases, Beijing Tiantan Hospital, Capital Medical University, Beijing, China; ^5^Department of Neurology, The Second Affiliated Hospital of Xiamen Medical College, Xiamen, China

**Keywords:** constipation, elderly, epidemiology, prevalence, risk factors

## Abstract

**Purpose:**

To investigate the epidemiological characteristics of constipation in people aged 65 years and older in several regions of China.

**Patients and Methods:**

A cross-sectional study based on a cluster sampling design was conducted in four cities of China: Tianjin, Xiamen, Cangzhou and Harbin. A total of 5,222 cases (age ≥ 65 years) were recruited, and the survey was conducted *via* centralized and household questionnaires that included the following: basic demographic characteristics such as sex, age, education, marital status, living status and occupation; social activities; duration of sleep at night; duration of menstruation and delivery times (in females); and if the participant had constipation symptoms, the severity of constipation. Constipation was diagnosed according to the Rome IV criteria.

**Results:**

Of the 5,222 participants, 919 were diagnosed with constipation. The prevalence of constipation was 17.60% in elderly people ≥65 years old. Prevalence increased with age and was significantly higher in females than males (*P* < 0.05). Prevalence was lower in the manual compared to the non-manual worker group, and significantly increased with decreasing duration of night sleep (*P* < 0.05). Older age, female sex and shorter sleep duration at night were risk factors for constipation in elderly people.

**Conclusion:**

The prevalence of constipation in the elderly people in four cities of China was 17.60%, and was significantly affected by age, sex and sleep duration at night.

## Introduction

Constipation is one of the most common functional gastrointestinal (GI) disorders and has the following symptoms: lumpy or hard stools, difficulty in defecation and decreased defecation frequency (1). It is a heterogeneous disorder with multiple causes, including dysfunction of intestinal motility, visceral sensitivity, anorectal musculature and the enteric nervous system (1, 2). The prevalence of constipation in the general population worldwide ranges from 0.7 to 79% (median 16%) (2). Constipation can be divided into two main categories: primary and secondary. Primary constipation is further divided into three main types: functional, outlet dysfunction, and slow transit constipation. Secondary constipation may be caused by dietary and exercise patterns, disease processes and adverse effects of medication (3, 4).

In recent years, constipation in elderly people has become a worldwide problem because of the characteristics of this population, such as decreased social activity, psychological disorders, pelvic floor aging, co-morbidity and effects from multiple drug use (5). Constipation has a major negative impact on quality of life relevant to both physical and emotional wellbeing and poses a large economic burden (4, 6–8), because constipation not only causes pain in elderly people but also leads to many complications such as hemorrhoids, fecal impaction, bowel perforations, fecal incontinence, rectal prolapse, volvulus, and excessive perineal or inadequate perineal descent (3, 4, 9). The therapies that can eliminate constipation include change of lifestyle and diet, alleviation of risk factors, over-the-counter or prescription laxatives and enemas. Nevertheless, only 22.2% of patients seek medical advice (10). It was reported that only 15.7–48.0% of patients use laxatives to treat their symptoms and 19.0% of patients do not proceed with any intervention (2).

There have been many studies on the prevalence of constipation, but the results differ greatly and there are few multicenter epidemiological investigations for constipation in Chinese elderly people. Population-based studies have reported that the prevalence range of constipation in North America is 2–27%, with most clustering around 15% (11, 12), while the prevalence of constipation in China is 3–11%, with prevalence of 13–32.6% in elderly people (5, 10, 13). Previous studies have shown that constipation prevalence is significantly affected by such factors as age, sex, occupation type, education, dietary habits, physical exercise and past history of other related illnesses (2, 5, 10, 13, 14). However, there are limited studies concerning the effects of sleep, menstruation and delivery on constipation. The aim of this study was to investigate the epidemiological characteristics of constipation in people aged 65 years and older in several regions of China and to identify the associated factors so that new preventive measures and therapeutic strategies can be applied.

## Materials and Methods

### Research Subjects

A cross-sectional study based on an epidemiological survey of dementia in elderly people (≥65 years old) in four cities in China was conducted during June–October 2019. Based on the cluster sampling design, we recruited samples from the communities of Tianjin, Xiamen, Cangzhou and Harbin cities, located in the north, northeast and south regions of China, constituting the baseline population structure data of China.

Based the multistage cluster sampling design, we considered geographical region, economic development level and population size according to 2010 census data and selected the four cities as centers. In the second stage, we selected a total of four urban areas and three towns for which the per capita gross domestic product (GDP) was closest to the median GDP of urban and rural populations. Finally, a total of nine communities and 69 villages were selected from the selected urban areas and towns.

All participants included were 65 years or older, were listed in the census of the community registry office and had lived in the community for at least 1 year preceding the survey date. Those listed in the census but institutionalized were not included in the study. Subjects who refused to participate, were untraceable, had a life-threatening illness, were deceased, or were unable to participate in the assessment because of inadequate hearing, speaking or vision were excluded. The protocol for this study was reviewed and approved by the Ethics Committee at Tianjin Huanhu Hospital. Informed consent was obtained from each subject either directly or from his or her guardian.

### Research Method

The survey was conducted *via* centralized and household questionnaires that included the following: basic demographic characteristics such as sex, age, education, marital status, living status and occupation; social activities; duration of sleep at night; duration of menstruation and delivery times in females; and, if the participant had constipation symptoms, the severity of constipation. Prior to conducting this survey, a pilot study surveyed one village to test the epidemiological methods. The research team was composed of clinicians and assistants who were trained for 1 week and unified the survey methods and standards. The questionnaire survey was conducted face to face with the assistance and coordination of community workers. The survey forms were filled manually by the research team, and the questionnaires were reviewed and administered by qualified and experienced neurologists with the assistance of gastroenterologists who all underwent the same training at Tianjin Huanhu Hospital in Tianjin, China. All research teams and experts received the same 1-week training on collecting information, neuropsychiatric scale assessments and diagnosis, and participated in a retraining course every 2 months.

The Rome IV criteria for constipation were taken as the standard reference with the following constipation assessment indicators. These included two or more defecation difficulties of the following: straining, having lumpy or hard stools, having sensation of incomplete evacuation, having sensation of anorectal obstruction/blockage, needing manual maneuvers to facilitate defecation more than one quarter of times or having fewer than three spontaneous bowel movements per week. The criteria were fulfilled for the last 3 months, with symptom onset at least 6 months prior to diagnosis (1). The participants who met the above criteria for constipation were asked about the severity of constipation, which was classified into four grades according to the frequency of constipation symptoms and interference in daily life: occurs rarely, sometimes, usually or always and interferes with daily life, represented by values of 1, 2, 3 and 4, respectively (15).

### Statistical Method

All analyses were performed with SPSS version 22.0. The prevalence of constipation was estimated for the entire study population and was also stratified by basic information such as age, sex, education years and so on. The measurement data were expressed as mean ± standard deviation. The enumeration data were expressed as constituent ratios (%) or rates (%), and comparisons were performed by Chi-square test. Binary logistic regression analysis was conducted to obtain the risk factors of constipation by taking age, sex, sleep duration at night and occupation as independent variables, which were significant in the Chi-square test. Two-tailed *P* < 0.05 was considered significant.

## Results

The total number of participants aged ≥65 years in these communities and villages was 5,461; however, due to refusal (*n* = 106), death (*n* = 10), migration (*n* = 12), hearing loss (*n* = 91), aphasia (*n* = 11) or other life-threatening illness (*n* = 9), a total of 5,222 completed the final interview. Overall, we studied 5,222 participants from four cities in China and the mean age of the sample was 72.26 ± 6.09 years.

### Demographic Characteristics of Participants

Of all participants, 2,269 were male (43.45%) with mean age of 72.57 ± 6.32 years and 2,953 were female (56.55%) with mean age of 72.02 ± 5.90 years. Regarding age of the participants, 2,139 were 65–69 years (40.96%), 1,467 were 70–74 years (28.09%), 849 were 75–79 years (16.26%), 758 were 80 years and older (14.52%) and nine had missing data (0.17%). The total participants were divided into groups by years of education: 1,218 had no schooling (23.32%), 2,408 received 1–6 years of education (46.11%), 927 received 7–9 years of education (17.75%), 409 received 10–12 years of education (7.83%), 242 received more than 12 years of education (4.63%) and 18 had missing data (0.34%).

Of all participants, 45 were single (0.86%), 3,939 were married (75.43%), 45 were divorced (0.86%), 1,156 were widowed (22.14%) and 37 had missing data (7.09%). Concerning living status, 3,599 lived with a spouse (68.92%), 1,115 lived without a spouse (21.35%) and 508 lived alone (9.73%). Regarding occupation of participants, 4,183 were manual workers (80.10%), 972 were non-manual workers (18.61%) and 67 had missing data (1.28%). Of all participants, 2,336 took part in social activities for 0–3 days every week (44.73%), 2,833 took part in social activities for 4–7 days every week (54.25%) and 53 had missing data (1.01%).

Participants were divided into groups by the duration of sleep at night: 31 cases slept <2 h every night (0.59%), 411 slept 2–4 h every night (7.87%), 1,331 slept 4–6 h every night (25.49%), 2,253 slept 6–8 h every night (43.15%), 953 slept more than 8 h every night (18.25%) and 243 had missing data (4.65%) (Table 1).

**Table 1:** Demographic characteristics of the study participants.

**Items**	**Num**	**Percentage**
All study participants	5,222	100%
**Sex**		
Male	2,269	43.45%
Female	2,953	56.55%
**Age group (y)**		
65–69	2,139	40.96%
70–74	1,467	28.09%
75–79	849	16.26%
≥80	758	14.52%
MD	9	0.17%
**Education (y)**		
0	1,218	23.32%
1–6	2,408	46.11%
7–9	927	17.75%
10–12	409	7.83%
>12	242	4.63%
MD	18	0.34%
**Marital status**		
Single	45	0.86%
Married	3,939	75.43%
Divorced	45	0.86%
Widowed	1,156	22.14%
MD	37	0.71%
**Living status**		
With spouse	3,599	68.92%
Without spouse	1,115	21.35%
Alone	508	9.73%
**Occupation**		
Manual worker	4,183	80.10%
Non-manual worker	972	18.61%
MD	67	1.28%
**Social activities**		
0–3 d/week	2,336	44.73%
4–7 d/week	2,833	54.25%
MD	53	1.01%
**Sleep duration**		
<2h	31	0.59%
2–4h	411	7.87%
4–6h	1,331	25.49%
6–8h	2,253	43.15%
≥8 h	953	18.25%
MD	243	4.65%

*MD, Missing data*.

The total female participants were divided into groups by the duration of menstruation: 183 cases had duration of <25-years (6.2%), 343 had 25–29-years (11.6%), 1,143 had 30–34-years (38.7%), 998 had more than 35-years (33.8%) and 286 had missing data (9.7%). In addition, the total female participants were divided into groups by delivery times: 2,277 cases (77.1%) had three deliveries or less, 568 (19.2%) had more than three deliveries and 108 had missing data (3.7%).

### Prevalence of Constipation

**Figure 1:**
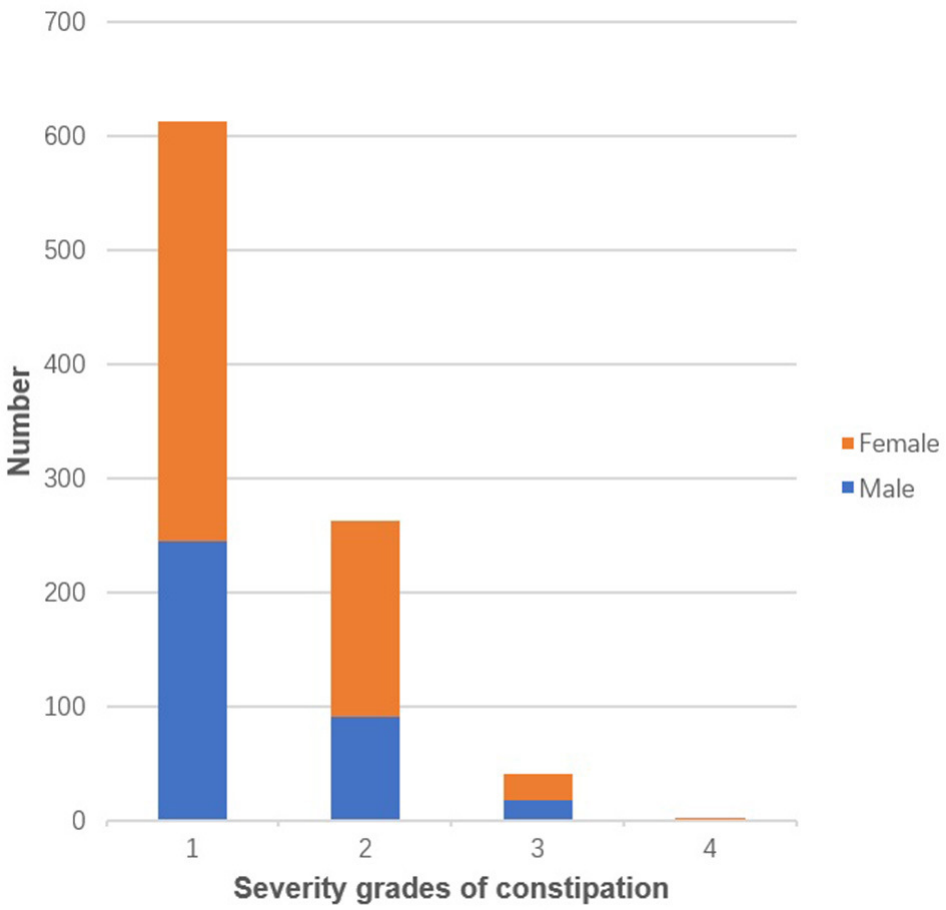
The number of different severity grades of constipation.

**Figure 2:**
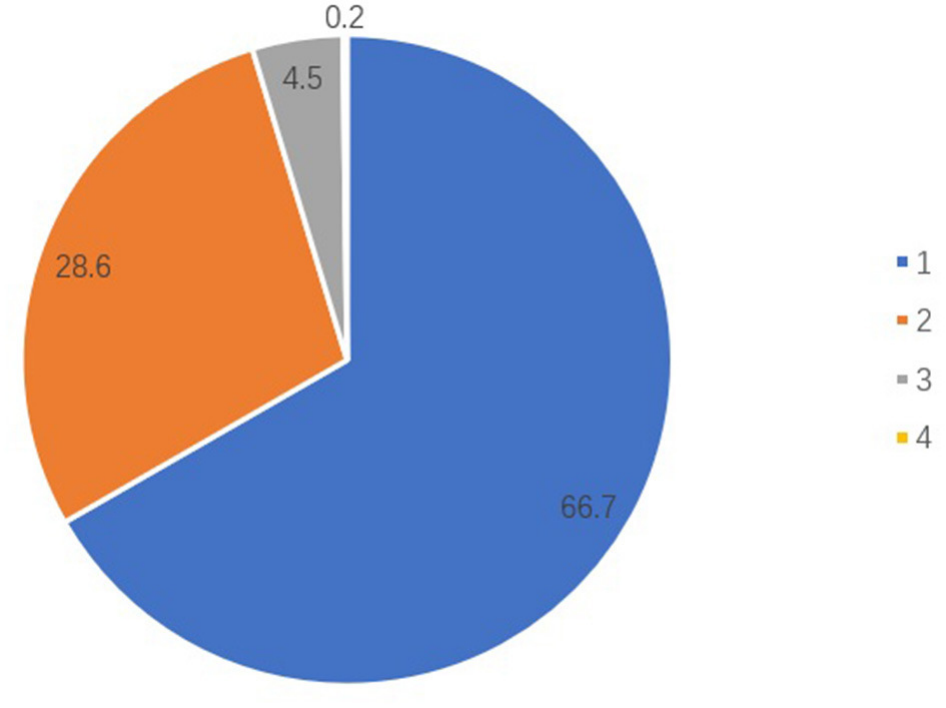
The proportion of different severity grades in people with constipation.

Of all participants, 919 were diagnosed with constipation, making a total prevalence rate of 17.6% (Table 1). The number of people with severity grades of 1, 2, 3 and 4 were 613, 263, 41 and 2, respectively (Figure 1); in people with constipation, the proportions of these different severity grades were 66.7, 28.6, 4.5 and 0.2%, respectively (Figure 2). The proportion decreased with increasing severity grades.

### Prevalence of Constipation According to Different Characteristics of Respondents

The prevalence of constipation was significantly higher (χ2 = 12.030, *P* = 0.001) in the female (19.2%) than the male group (15.5%). There were 299 (14.0%), 263 (17.9%), 159 (18.7%) and 197 (26.0%) cases with constipation in the respective age groups proceeding from younger to older. The prevalence of constipation significantly increased with age (χ2 = 56.959, *P* < 0.001). Regarding occupation, there were 713 cases (17.0%) and 194 cases (20.0%) with constipation in the respective groups. The prevalence of constipation was significantly lower in the manual compared to non-manual worker group (χ2 = 4.618, *P* = 0.032). Regarding the duration of sleep at night, there were 10 (32.3%), 91 (22.1%), 280 (21.0%), 361 (16.0%) and 139 cases (14.6%) with constipation in the respective groups, with prevalence significantly increasing with decreasing sleep duration at night (χ2 = 30.951, *P* < 0.001). In women, there were 422 cases (18.5%) with constipation in those with three deliveries or less and 127 (22.4%) for those with more than three deliveries. The prevalence of constipation was significantly higher in the latter group (χ2 = 4.273, *P* = 0.039). In addition, there were no significant differences in constipation prevalence among the education, marital status, living status, social activity and menstruation duration groups (*P* > 0.05) (Table 2).

**Table 2:** Comparison of the prevalence of constipation between different groups.

**Group**	**With**	**Without**	**χ^2^**	***P***
	**constipation**	**constipation**		
	**(percentage)**	**(percentage)**		
**Sex**			12.03	**0.001**
Male	352 (15.5)	1,917 (84.5)		
Female	567 (19.2)	2,386 (80.8)		
**Age group (y)**			56.959	**<0.001**
65–69	299 (14.0)	1,840 (86.0)		
70–74	263 (17.9)	1,204 (82.1)		
75–79	159 (18.7)	690 (81.3)		
≥80	197 (26.0)	561 (74.0)		
MD	–	–		
**Education (y)**			6.000	0.199
0	219 (18.0)	999 (82.0)		
1–6	432 (17.9)	1,976 (82.1)		
7–9	146 (15.7)	781 (84.3)		
10–12	67 (16.4)	342 (83.6)		
>12	53 (21.9)	189 (78.1)		
MD	–	–		
**Marital status**			1.813	0.612
Single	9 (20.0)	36 (80.0)		
Married	691 (17.5)	3,248 (82.5)		
Divorced	5 (11.1)	40 (88.9)		
Widow	211 (18.3)	945 (81.7)		
MD	–	–		
**Living status**			0.105	0.949
With spouse	632 (17.6)	2,967 (82.4)		
Without spouse	195 (17.5)	920 (82.5)		
Alone	92 (18.1)	416 (81.9)		
**Occupation**			4.618	**0.032**
Manual worker	713 (17.0)	3,470 (83.0)		
Non-manual worker	194 (20.0)	778 (80.0)		
MD	–	–		
**Social activities**			0.161	0.688
0–3 d/week	414 (17.7)	1,922 (82.3)		
4–7 d/week	490 (17.3)	2,343 (82.7)		
MD	–	–		
**Sleep duration**			30.951	**<0.001**
<2h	10 (32.3)	21 (67.7)		
2–4h	91 (22.1)	320 (77.9)		
4–6h	280 (21.0)	1,051 (79.0)		
6–8h	361 (16.0)	1892 (84.0)		
≥8 h	139 (14.6)	814 (85.4)		
MD	–	–		
**Duration of menstruation in females (y)**			2.145	0.543
<25	30 (16.4)	153 (83.6)		
25–29	60 (17.5)	283 (82.5)		
30–34	220 (19.2)	923 (80.8)		
≥35	201 (20.1)	797 (79.9)		
MD	–	–		
**Delivery times in females**			4.273	**0.039**
≤ 3	422 (18.5)	1,855 (81.5)		
>3	127 (22.4)	441 (77.6)		
MD	–	–		

*MD, Missing data. Bold values represent P value less than 0.05 to make the results distinct and obvious*.

### Logistic Regression Analysis of Risk Factors for Constipation

Binary logistic regression analysis was conducted to determine the risk factors of constipation by taking age, sex, sleep duration and occupation as independent variables, which were significant in the Chi-square test. The logistic regression chose forward stepwise regression method. The results showed that females had a significantly increased risk of constipation compared to males [odds ratio (OR) = 1.238, 95% confidence interval (CI) 1.064–1.441, *P* = 0.006]. Compared to the youngest group (65–69 years), increasing age significantly increased the risk of constipation (70–74 years: OR = 1.350, 95% CI 1.122–1.624, *P* = 0.001; 75–79 years: OR = 1.421, 95% CI 1.144–1.765, *P* = 0.002; ≥80 years: OR = 2.181, 95% CI 1.767–2.692, *P* < 0.001). Compared to the longest sleep duration group (≥8 h), too short a sleep duration at night (<6 h) significantly increased the risk of constipation (4–6 h group: OR = 1.534, 95% CI 1.225–1.921, *P* < 0.001; 2–4 h group: OR = 1.517, 95% CI 1.126–2.043, *P* = 0.006; <2 h group: OR = 2.395, 95% CI 1.095–5.239, *P* = 0.029) (Table 3). The results revealed that old age, female sex and shorter sleep duration at night were risk factors for constipation.

**Table 3:** Logistic regression analysis of risk factors for constipation.

**Group**	**OR**	**95% OR**	**95% OR**	***P***
		**Lower limit**	**Upper limit**	
**Sex**				
Male	1	–	–	
Female	1.238	1.064	1.441	**0.006**
**Age (y)**				**<0.001**
65–69	1	–	–	
70–74	1.35	1.122	1.624	**0.001**
75–79	1.421	1.144	1.765	**0.002**
≥80	2.181	1.767	2.692	**<0.001**
**Sleep duration**				**<0.001**
<2h	2.395	1.095	5.239	**0.029**
2–4h	1.517	1.126	2.043	**0.006**
4–6h	1.534	1.225	1.921	**<0.001**
6–8h	1.133	0.915	1.402	0.253
≥8 h	1	–	–	
**Occupation**				
Non-manual worker	1	–	–	
Manual worker	0.858	0.715	1.031	0.101

*Bold values represent P value less than 0.05 to make the results distinct and obvious*.

## Discussion

In the four studied cities, the total prevalence of constipation was 17.6% in people 65 years and older and increased with age. This is similar to the study by Chu et al., who reported a prevalence rate of 18.1% for constipation in the elderly population in China (10). Yang et al. showed that the prevalence of constipation among elderly people, over 60 years old, in the Beijing region was 13% (13), which was lower than our finding. There are some likely reasons for this disparity, such as age of participants, regional difference and different assessments of constipation. According to a review by Mugie et al., the prevalence of constipation in the general population worldwide is in the ranges of 0.7–79% (median 16%), with median prevalence in the general population of 19.2% in Europe and 19.7% in Oceania, indicating higher rates than in this study (2). The prevalence of constipation may vary with such factors as geographical areas, diagnostic criteria, age distributions of study populations, methods of investigation, socioeconomic conditions and diverse cultural characteristics. Regardless, the prevalence of constipation in this study seemed to be lower than in Western countries (2, 10). This may be due to higher diet quality, more physical activity and a different life style in China (16).

In our study, the prevalence of constipation increased with age and older age was one of the risk factors of constipation, which is consistent with other studies (2, 5, 10, 11, 14, 17). There are some likely primary causes that accompany aging. It is well-known that there are changes in mechanical properties (e.g., loss of plasticity and compliance), macroscopic structure (e.g., diverticulosis) and control of the pelvic floor impact bowel structure and function with advancing age (18, 19). Colonic transit time slows with aging, with propulsive efficacy of the colon decreasing (2, 18). Regarding the enteric nervous system, age-related loss of colonic neurons and changes in the morphology of the myenteric plexus of the human colon may cause slower colonic transit time or other physiological changes. Also, pelvic floor dysfunction is frequent in the elderly, especially women, which manifests as paradoxic contractions or inadequate relaxation of the pelvic floor muscles, or inadequate propulsive forces during attempted defecation (18, 19). The secondary causes of constipation include illness and multiple medication use, which are more common in elderly people. Several diseases including diabetes, dementia, Parkinson's disease, rheumatoid arthritis, stroke and multiple sclerosis, and various medications such as anticholinergic drugs, antiparkinsonism drugs, calcium-channel blockers, non-steroidal anti-inflammatory drugs, antihistamines and antihypertensives can increase occurrence of constipation (2–4). Elderly people are more prone to these diseases and to take medicine, which may increase the risk of constipation. Furthermore, elderly people may be particularly sensitive to the adverse effects of many medications and are more likely than their younger counterparts to manifest symptoms of constipation because of age-related physiological changes (4). In addition, elderly people often have a decreased appetite with lower fiber and fluid intake, and less physical exercise because of poor physical condition, which are high risks for constipation (3).

This study showed that the prevalence of constipation was lower in the manual compared to the non-manual worker group, consistent with other studies (5, 14). The non-manual group has a more sedentary lifestyle, which means less physical exercise, higher risk for obesity and greater mental stress. Because these factors are related to a trend toward increased prevalence of constipation, it is not hard to understand the result (2).

The prevalence of constipation was higher in the female than the male group, consistent with existing studies (2, 5, 10, 11, 17), which is related to female-specific physiological characteristics. It is known that men have a greater skeletal muscle mass and a longer anal sphincter compared to women, and that the increased anterolateral abdominal wall musculature in men may allow for increased abdominal pressure during defecation. Men also have a higher sphincter resting pressure and squeeze pressure than women (20). In addition, this result may be attributed to unique hormonal fluctuations in females. Receptors of female sex hormones such as estrogen and progesterone are expressed throughout the GI tract. These hormones can influence visceral sensitivity and visceromotor, and slow gastric emptying and intestinal transit (21, 22). Under the effect of progesterone during the luteal phase of the menstrual cycle or gestation period, the contraction of intestinal smooth muscle is inhibited and the transit time of the small intestine and colon is increased, which cause difficulty in defecation (10, 23, 24). This study showed that the prevalence of constipation increased with longer menstruation time, although this was not significant, and may be due to the longer effect of female sex hormones. Menopause means decreasing levels of estrogen and progesterone. The study by Huang et al. found that women in the premenopausal period were more likely to suffer from constipation than those in the postmenopausal period (14), which may support the views above. Moreover, menopause has been shown to affect muscle strength, tissue elasticity and resilience to load bearing in the pelvis (19, 20). Dyssynergic defecation is associated with pelvic floor dysfunction and particularly with paradoxical contraction or insufficient relaxation of the levator ani, anal sphincter and abdominal wall muscles. However, the role of menopause in pelvic floor dysfunction is unclear. There may be involvement of hormones such as estrogen, progesterone, catecholamines and prostaglandins (25, 26). This study showed that the prevalence of constipation was higher in females who had more deliveries. Damage to pelvic floor muscles and nerves during delivery cause pelvic floor dysfunction, which is frequent in older women and increases the risk of constipation (10, 19, 20, 27). All these factors may explain the higher prevalence of constipation in females and why female sex is a risk factor for constipation.

The prevalence of constipation increased with decreasing sleep duration at night, and logistic regression analysis revealed that shorter sleep duration (<6 h) increased the risk of constipation in elderly people. This is consistent with some previous studies in which participants were younger (14, 28–30). Tam et al. found that nighttime sleep duration <7 h was an independent risk factor for constipation among elementary school children in Hong Kong (31). Some studies indicated that poor sleep increased the risk of functional constipation among college students (32, 33). A study of functional GI disorders and sleep duration found that functional constipation was associated only with decreased sleep rather than other sleep disorders like drowsiness (34). Too short a duration of night sleep is likely caused by sleep disturbances, such as difficulties falling asleep, difficulties maintaining sleep during the night, waking up too early in the morning and non-restorative sleep (35). Some studies have shown that sleep disturbances increase the prevalence of constipation and cause more severe constipation symptoms (14, 28, 36–38). Jiang et al. hypothesize that sleep disturbances may result in dysfunction of the anal sphincter, enhance pelvic floor muscle tone and cause anorectal contradictory movements or paresthesia, which may aggravate constipation symptoms. There may also be specific neuroendocrine regulatory mechanisms for the visceral hypersensitivity-related symptoms which need to be explored (28). Sleep disturbances may disrupt the communication between areas in the brain (insula, anterior cingulate cortex, thalamus and prefrontal cortex) and the GI tract, causing GI autonomic dysfunction (39). Additionally, there may be an inflammatory cytokine mechanism, such as tumor necrosis factor and interleukin−1 and −6 (33, 39). Patients with sleep disturbances suffer from more severe anxiety and depression associated with constipation and this leads to negative effects by modulating the brain–gut axis and affecting gut motility (28, 29, 31, 38). Sleep disturbances may aggravate constipation symptoms separately or in combination with anxiety and depression (28, 36).

As a part of this multicenter study, samples from multiple regions in China were collected, which tended to eliminate regional differences, and the numerous samples reduced error. However, our results may not reflect the situation of all regions in China and may be more representative of only the four investigated regions. In addition, there have been few studies examining the effect of sleep duration on constipation, while the present study revealed that the duration of sleep at night was one risk factor for constipation. Owing to some limitations of the investigation, other factors such as dietary habits, physical exercise and illness and medication histories were not considered in this study, which may have affected the occurrence of constipation. Moreover, the result depended on the recall of participants, which might have caused recall bias. Future studies will further expand the geographical coverage, increase the sample size and use better designs of questionnaires to accurately reflect the prevalence of constipation in elderly people in China and explore more of the risk factor.

## Conclusion

The prevalence of constipation in elderly people in four cities of China was 17.60%, and it is significantly affected by age, sex and sleep duration at night. Constipation in elderly people may be managed by controlling these risk factors, which should be of interest to physicians.

## Data Availability Statement

The raw data supporting the conclusions of this article will be made available by the authors, without undue reservation.

## Ethics Statement

The protocol for this study was reviewed and approved by the Ethics Committee at Tianjin Huanhu Hospital [2019-40]. Informed consent was obtained from each subject either directly or from his or her guardian. The patients/participants provided their written informed consent to participate in this study.

## Author Contributions

XD, SL, and PJ have designed, implemented, and wrote the article. XW, JG, WH, HZ, and YS have helped to implemented the article and collected the cases. YJ and JN have directed the article. All authors contributed to the article and approved the submitted version.

## Funding

This work was supported by the National Natural Science Foundation of China (Grant Number 82171182), Science and Technology Project of Tianjin Municipal Health Committee (Grant Numbers ZC20121 and KJ20048), and Tianjin Key Medical Discipline (Specialty) Construction Project.

## Conflict of Interest

The authors declare that the research was conducted in the absence of any commercial or financial relationships that could be construed as a potential conflict of interest.

## Publisher's Note

All claims expressed in this article are solely those of the authors and do not necessarily represent those of their affiliated organizations, or those of the publisher, the editors and the reviewers. Any product that may be evaluated in this article, or claim that may be made by its manufacturer, is not guaranteed or endorsed by the publisher.
